# BI 1015550 is a PDE4B Inhibitor and a Clinical Drug Candidate for the Oral Treatment of Idiopathic Pulmonary Fibrosis

**DOI:** 10.3389/fphar.2022.838449

**Published:** 2022-04-20

**Authors:** Franziska Elena Herrmann, Christian Hesslinger, Lutz Wollin, Peter Nickolaus

**Affiliations:** Boehringer Ingelheim Pharma GmbH & Co. KG, Biberach an der Riss, Germany

**Keywords:** PDE4B, IPF, lung fibrosis, phosphodiesterase, cAMP, ILDs

## Abstract

The anti-inflammatory and immunomodulatory abilities of oral selective phosphodiesterase 4 (PDE4) inhibitors enabled the approval of roflumilast and apremilast for use in chronic obstructive pulmonary disease and psoriasis/psoriatic arthritis, respectively. However, the antifibrotic potential of PDE4 inhibitors has not yet been explored clinically. BI 1015550 is a novel PDE4 inhibitor showing a preferential enzymatic inhibition of PDE4B. *In vitro*, BI 1015550 inhibits lipopolysaccharide (LPS)-induced tumor necrosis factor-α (TNF-α) and phytohemagglutinin-induced interleukin-2 synthesis in human peripheral blood mononuclear cells, as well as LPS-induced TNF-α synthesis in human and rat whole blood. *In vivo*, oral BI 1015550 shows potent anti-inflammatory activity in mice by inhibiting LPS-induced TNF-α synthesis *ex vivo* and in Suncus murinus by inhibiting neutrophil influx into bronchoalveolar lavage fluid stimulated by nebulized LPS. In Suncus murinus, PDE4 inhibitors induce emesis, a well-known gastrointestinal side effect limiting the use of PDE4 inhibitors in humans, and the therapeutic ratio of BI 1015550 appeared to be substantially improved compared with roflumilast. Oral BI 1015550 was also tested in two well-known mouse models of lung fibrosis (induced by either bleomycin or silica) under therapeutic conditions, and appeared to be effective by modulating various model-specific parameters. To better understand the antifibrotic potential of BI 1015550 *in vivo*, its direct effect on human fibroblasts from patients with idiopathic pulmonary fibrosis (IPF) was investigated *in vitro*. BI 1015550 inhibited transforming growth factor-β-stimulated myofibroblast transformation and the mRNA expression of various extracellular matrix proteins, as well as basic fibroblast growth factor plus interleukin-1β-induced cell proliferation. Nintedanib overall was unremarkable in these assays, but interestingly, the inhibition of proliferation was synergistic when it was combined with BI 1015550, leading to a roughly 10-fold shift of the concentration–response curve to the left. In summary, the unique preferential inhibition of PDE4B by BI 1015550 and its anticipated improved tolerability in humans, plus its anti-inflammatory and antifibrotic potential, suggest BI 1015550 to be a promising oral clinical candidate for the treatment of IPF and other fibro-proliferative diseases.

## Graphical Abstract

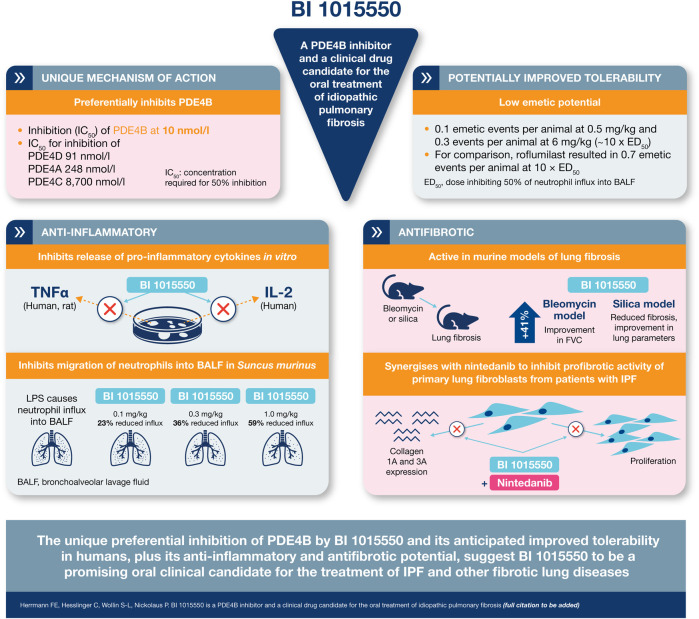

## Introduction

Interstitial lung diseases (ILDs) comprise a heterogeneous group of lung diseases affecting the interstitium, distinct from obstructive airway diseases such as asthma or chronic obstructive pulmonary disease (COPD). The most extensively studied ILD is idiopathic pulmonary fibrosis (IPF), which is characterized by progressive pulmonary fibrosis. Non-IPF ILDs may include connective tissue disease-related ILDs such as those related to rheumatoid arthritis and other autoimmune diseases, systemic sclerosis (SSc), and polymyositis/dermatomyositis, and ILDs related to chronic sarcoidosis, chronic hypersensitivity pneumonitis, idiopathic non-specific interstitial pneumonia, and exposure-related diseases such as asbestosis and silicosis (Cottin et al., 2019; Kolb and Vašáková, 2019). Up to 40% of patients with these ILDs may develop a progressing fibrotic phenotype.

Progressing fibrosing ILDs are associated with high mortality, with median post-diagnosis survival in patients with IPF estimated at 2–5 years (Raghu et al., 2014). Progression of fibrosing ILD is reflected in various parameters, including decline in pulmonary function, decrease in exercise capacity, deterioration in quality of life, worsening of cough and dyspnea, acute exacerbations, and increase of morphologic abnormalities (Cottin et al., 2019; Kolb and Vašáková, 2019). In patients with IPF, forced vital capacity (FVC) is a well-established predictor of mortality, and acute exacerbations are associated with very high mortality. Although corticosteroids and/or immunosuppressive drugs are sometimes used off-label to treat progressing fibrosing ILDs, currently the only approved treatments to slow disease progression in IPF are nintedanib, a tyrosine kinase inhibitor, and pirfenidone, a pyridone with an unknown mechanism of action (Richeldi et al., 2018). Nintedanib has been approved in the US since 2014 (U.S. Food and Drug Administration, 2020), and in Europe and Japan since 2015 (European Medicines Agency, 2021b), while pirfenidone was approved in Japan in 2008 (BioBusiness Briefs, 2008), in Europe in 2011 (European Medicines Agency, 2021a), and in the US in 2014 (U.S. Food and Drug Administration, 2019). Lung transplantation is the only potentially curative treatment for IPF, and the medical need in IPF and other progressive fibrosing ILDs remains high.

With respect to IPF pathophysiology, there has been a paradigm shift in recent years from a chronic inflammatory disorder to a primarily fibrotic disease. The current paradigm of disease pathogenesis involves sustained alveolar epithelial micro-injury, followed by a disordered repair and wound-healing response. This is characterized by uncontrolled activation of lung fibroblasts and differentiation to myofibroblasts, resulting in excessive extracellular matrix deposition, scarring of lung parenchyma, and (probably irreversible) loss of pulmonary function (Sgalla et al., 2018; Spagnolo et al., 2018). The wound-healing process includes an inflammatory phase, with the involvement of inflammatory cells (in particular macrophages, monocytes, neutrophils, and T lymphocytes) and increased levels of cytokines and growth factors, creating a biochemical environment supporting chronic tissue remodeling. Both drugs effective in the treatment of IPF, nintedanib and pirfenidone, have been shown to have antifibrotic and anti-inflammatory activity (Heukels et al., 2019).

Phosphodiesterases (PDEs) mediate the hydrolysis of the second messengers, cyclic adenosine monophosphate (cAMP) or cyclic guanosine monophosphate (cGMP). PDEs are coded by 11 gene superfamilies containing multiple genes (coding for subtypes A, B, C, etc.) that also give rise to alternative mRNA-splicing variants leading to approximately 100 PDE isoforms (Azevedo et al., 2014).

The PDE4 subtypes A–D are encoded by different genes, PDE4A, B, C, and D, with post-translational processing resulting in N-terminal variant groups (long, short, and super-short form) according to the presence or absence of upstream conserved regions 1 and 2 (UCR1 or UCR2) N-terminal domains (Bolger, 1994; Beard et al., 2000). PDE signaling is highly compartmentalized as PDE4 subtypes can integrate into macromolecular complexes known as signalosomes. For example, in cardiac signalosomes, PDE4D5 interacts with β-arrestin, regulating receptor desensitization, and PDE4B regulates both L-type calcium channels and ryanodine receptor, which controls the process of calcium-induced calcium release (Bolger et al., 2003; Lehnart et al., 2005; Terrenoire et al., 2009; Beca et al., 2011; Leroy et al., 2011; Sin et al., 2011; Maurice et al., 2014). This presents an opportunity to develop subtype-specific PDE4 inhibitors for different conditions and maximize efficacy and tolerability profiles.

PDE4 has traditionally been implicated in the regulation of inflammation and the modulation of immunocompetent cells, and the three selective PDE4 inhibitors currently available support a beneficial role for PDE4 inhibitors in inflammatory and/or autoimmune diseases (Sakkas et al., 2017; Li et al., 2018). Evidence points to the involvement of a range of immune cells and inflammatory responses in IPF. The results of previous negative trials altering inflammation may suggest that inflammatory changes seen in IPF occur independently of the primary fibrotic remodeling process. Observations from human trials and experimental modeling indicate that changes may be needed to the “traditional” model of pulmonary fibrosis, with immune cells, such as macrophages and lymphocytes, modulating existing fibrotic responses (Desai et al., 2018; Heukels et al., 2019).

The first-in-class PDE4 inhibitor, oral roflumilast (Daliresp®, Daxas®), was approved by the U.S. Food and Drug Administration in 2011 to reduce the risk of COPD exacerbations in patients with severe COPD associated with chronic bronchitis and a history of exacerbations (U.S. Food and Drug Administration, 2013). Another compound, oral apremilast (Otezla®), was approved for the treatment of psoriatic arthritis and plaque psoriasis in 2014 (U.S. Food and Drug Administration, 2017). A third PDE4 inhibitor, crisaborole (Eucrisa®), was approved in 2016 for topical treatment of mild-to-moderate atopic dermatitis (U.S. Food and Drug Administration, 2016). None of these show any preferential enzymatic inhibition among the four PDE4 subtypes, A–D.

The general anti-inflammatory potential of PDE4 inhibition, as exemplified by roflumilast, is well established (Hatzelmann et al., 2010), and the use of PDE4 inhibitors in various inflammatory and immune-mediated diseases has been broadly investigated (Sakkas et al., 2017; Li et al., 2018). However, in the last decade, it has become increasingly clear that PDE4 may also play an important role in fibrosis, based on animal studies and on *in vitro* experiments evaluating the functional role of PDE4 inhibitors in fibroblasts. The attenuation of lung fibrosis by PDE4 inhibitors has been demonstrated under various experimental conditions, most widely in bleomycin-induced fibrosis in rodents. In rat models, rolipram was shown to inhibit fibrotic score, hydroxyproline content, and serum tumor necrosis factor-α (TNF-α) (Pan et al., 2009). In this initial study, the PDE4 inhibitor was administered from the beginning of bleomycin challenge, so it was not clear whether rolipram was primarily active due to inhibition of initial inflammation or inhibition of secondary fibrosis. A second early study in mice and rats, however, showed that oral roflumilast was active both in preventive and in therapeutic protocols in a dose-dependent manner (Cortijo et al., 2009). In lung extracts, roflumilast inhibited histologically assessed fibrosis, hydroxyproline content, and the mRNA expression of TNF-α, transforming growth factor-ß (TGF-ß), connective tissue growth factor (CTGF), α1 collagen, endothelin-1, and mucin 5ac. In bronchoalveolar lavage fluid (BALF), the levels of TNF-α, interleukin (IL)-13, TGF-ß, and mucin 5ac, the formation of lipid hydroperoxides, and the influx of inflammatory cells (e.g. neutrophils and macrophages) were inhibited. Besides fibrosis, right ventricular hypertrophy and vascular remodeling (pulmonary arteries) were positively influenced by roflumilast. The same group later also demonstrated that the metabolome associated with pulmonary fibrosis in bleomycin mice was modulated by roflumilast. Levels of the amino acids (AAs) glycine and proline, involved in collagen formation/structure, were lowered by roflumilast, while lung glutathione and plasma tetrahydrobiopterin were increased, suggesting an alteration in oxidative equilibrium by roflumilast (Milara et al., 2015). Another PDE4 inhibitor, cilomilast, was shown to inhibit late-stage lung fibrosis and tended to reduce collagen content in bleomycin mice, although no effect on TGF-ß1 and collagen type (Col) 1A1 expression was found (Udalov et al., 2010).

Improvement of lung fibrosis by PDE4 inhibition was not limited to the bleomycin model. In a murine model of lung fibrosis targeting type II alveolar epithelial cells in transgenic mice expressing the diphtheria toxin receptor under the control of the murine surfactant protein C promoter, roflumilast lowered lung hydroxyproline content and mRNA expression of TNF-α, fibronectin (FN), and CTGF (Sisson et al., 2018). Interestingly, roflumilast was active both in a preventive and in a therapeutic regimen, and under the latter conditions appeared to be therapeutically equi-effective to pirfenidone and nintedanib. Furthermore, in a mouse model of chronic graft-versus-host disease, lung fibrosis was attenuated by oral roflumilast (Kim et al., 2016). Roflumilast inhibited fibrosis, collagen deposition, hydroxyproline and TGF-ß1 content, cell infiltration, and expression of mRNA for IL-6 and IL-1ß. In addition, in the BALF inflammatory cells (macrophages, lymphocytes, neutrophils, and eosinophils), expression of mRNA for IL-6, IL-1ß, and monocyte chemotactic protein-1 was inhibited by roflumilast. In a rabbit tuberculosis model, pulmonary damage and fibrosis were shown to be inhibited by two PDE4 inhibitors from Celgene, CC-3052 (Subbian et al., 2011) and CC-11050 (Subbian et al., 2016). PDE4 inhibition improved antibiotic therapy and lung fibrosis by positively influencing collagen deposition and mRNA expression of various matrix metalloproteinases, including metalloproteinase 12.

Besides the lung, beneficial effects of PDE4 inhibition on fibrosis have been demonstrated in several other organs including skin, liver, kidney, and colon. For example, in various preclinical mouse models of SSc (bleomycin-induced, topoisomerase I and chronic graft-versus-host disease), skin fibrosis was inhibited by rolipram and apremilast (Maier et al., 2017). This group did not find direct inhibitory effects of PDE4 inhibition on the release of profibrotic cytokines (IL-6, IL-13, TGF-ß1/ß2) in fibroblasts and M2 macrophages purified from peripheral blood of patients with SSc, which may be due to the lack of an exogenous cAMP trigger under the experimental conditions used. In a model of unilateral ureteral obstruction-induced obstructive nephropathy in mice, rolipram was shown to inhibit renal interstitial fibrosis (Ding et al., 2018). In mouse primary tubular epithelial cells *in vitro*, TGF-ß upregulated PDE4A/B, and rolipram inhibited TGF-ß-induced damage, FN expression, and deficiency of mitochondrial biogenesis (Ding et al., 2018). Roflumilast inhibited diethylnitrosamine-induced liver fibrosis, hydroxyproline deposition, and TGF-ß1 expression in rats (Essam et al., 2019). Similarly, rolipram inhibited collagen deposition, α-smooth muscle actin (α-SMA) staining, and mRNA expression, as well as the expression of TGF-ß1 mRNA and TNF-α protein, in a bile duct ligation-induced hepatic fibrosis model in rats, with upregulation of PDE4A, B, and D (Gobejishvili et al., 2013). In hepatic stellate cells *in vitro*, rolipram inhibited mRNA expression of α-SMA and Col1A2 (Gobejishvili et al., 2013). With respect to colonic tissue, rolipram inhibited collagen and TGF-ß1 in a model of trinitrobenzene sulfonic acid-induced colitis in rats (Videla et al., 2006), and apremilast inhibited fibrosis in colon, collagen deposition, and the expression of genes related to fibrosis in a model of dextran sulfate sodium-induced colitis ulcerosa in mice (Li et al., 2019). In a murine cecal abrasion model, rolipram inhibited fibrotic reactions, indicating that PDE4 inhibition has the potential to prevent postoperative intra-abdominal adhesions (Eser et al., 2012). Adhesions are assumed to result from laparotomy by abnormal healing. In support of this assumption, rolipram has been shown to be active in a subcutaneous or intraperitoneal polyether-polyurethane sponge implant model in mice by inhibiting intra-implant collagen and TGF-ß1 deposition (Mendes et al., 2009). Thus, in various animal models, the beneficial impact of selective PDE4 inhibition on fibrosis has been proven, most extensively in the lung but also in several other organs. While the specific target(s) of PDE4 inhibitors in fibrotic diseases are largely unknown, it is tempting to speculate that they act either indirectly via inhibition of pro-inflammatory cells and mediators, and/or directly by inhibiting the typical effector cells (fibroblasts, myofibroblasts) mediating fibrosis.

The direct modulation of various fibroblast functions by PDE4 inhibitors has been demonstrated in fibroblast cell lines of human origin. Kohyama et al. demonstrated a direct impact of PDE4 inhibitors on fibroblasts *in vitro* (Kohyama et al., 2004). In human fetal lung fibroblasts (HFL-1), rolipram and cilomilast inhibited FN-induced chemotaxis and contraction of collagen gels. Inhibition of fibroblast function by prostaglandin E_2_ (PGE_2_) was shifted to the left in the presence of PDE4 inhibitors, and the inhibition of endogenous PGE_2_ by indomethacin diminished their effects (Kohyama et al., 2002). The inhibition of the fibroblast cell line HFL-1 functions by cilomilast could be modulated by cytokines like IL-1ß (which upregulated PGE_2_ and shifted the cilomilast curve to the left) or IL-4 (which downregulated PGE_2_ and shifted the cilomilast curve to the right). The inhibition of HFL-1 functions (FN-stimulated chemotaxis and collagen gel contraction) by rolipram and roflumilast was dependent on cyclooxygenase-2 expression and subsequent PGE_2_ synthesis (Kohyama et al., 2002). In addition, TGF-ß1-stimulated FN release was inhibited by the PDE4 inhibitors, paralleled by stimulation of PGE_2_ release as a positive feedback mechanism (Togo et al., 2009). In another human fetal lung fibroblast strain (GM06114), roflumilast N-oxide, the active metabolite of roflumilast, in the presence of PGE_2_ was shown to inhibit intercellular adhesion molecule-1 and eotaxin release stimulated by TNF-α, proliferation stimulated by basic fibroblast growth factor (bFGF) plus IL-1ß, as well as TGF-ß1-induced α-SMA, CTGF, and FN mRNA expression in the presence of IL-1ß (Sabatini et al., 2010). Importantly, IL-1ß upregulated PDE4 activity.

In normal human lung fibroblasts (NHLFs), the impact of PDE4 inhibitors has been described in several publications. TGF-ß-induced fibroblast-to-myofibroblast conversion assessed by α-SMA expression was shown to be inhibited by piclamilast in the presence of PGE_2_ (Dunkern et al., 2007). In subsequent papers, the same group showed the inhibition of IL-1ß plus bFGF-stimulated fibroblast proliferation by piclamilast and the importance of cyclooxygenase-2 and PGE_2_ (Selige et al., 2010). By using PDE4 subtype-specific siRNA, the involvement of PDE4B and PDE4A in this response, as well as the involvement of PDE4B and PDE4D in TGF-ß-induced α-SMA expression, were shown (Selige et al., 2011). The importance of a cAMP trigger for the modulation of fibroblast functions by PDE4 inhibition was corroborated by the inhibition by roflumilast of TGF-ß1-induced CTGF mRNA and α-SMA protein expression, and FN in the presence of the long-acting ß2-adrenergic agonist indacaterol (Tannheimer et al., 2012). In addition, various other NHLF responses (collagen synthesis, proliferation, reactive oxygen species and F2-isoprostane formation, NADPH oxidase 4 expression) stimulated by bleomycin or 8-epi-PGF_2α_ were inhibited by roflumilast N-oxide (Vecchio et al., 2013). In yet another human lung fibroblast model (WI-38), TGF-ß-induced mRNA expression of collagen α1, CTGF, and FN in the presence of the adenyl cyclase activator forskolin was inhibited by roflumilast and another PDE4 inhibitor (compound 1) (Sisson et al., 2018), as well as a dual-selective PDE4/5 inhibitor (compound A) (Muraki et al., 2019).

Thus, a multitude of *in vitro* studies indicate that PDE4 inhibitors are able to directly inhibit various fibroblast functions where either an endogenous or an exogenous cAMP trigger is available in the test system. While the availability of cAMP agonists may be limited under artificial *in vitro* conditions, it can be expected that in diseased (inflammatory, fibrotic) tissues, cAMP agonists like PGE_2_, adenosine, histamine, or adrenalin may be formed. The importance of PDE4 under such conditions may be further enhanced by upregulation of PDE4 activity by cytokines such as IL-1ß.

The modulation of another interesting aspect of fibrosis, epithelial-mesenchymal transition, was addressed in the TGF-ß1-stimulated A549 human alveolar epithelial cell line *in vitro*. TGF-ß1 stimulated the upregulation of PDE4 subtypes (PDE4A and 4D), and rolipram and siRNA against PDE4A and D inhibited epithelial-mesenchymal transition changes like FN and collagen mRNA expression, although not α-SMA mRNA (Kolosionek et al., 2009).

Emesis is a known class-related side effect of PDE4 inhibitors, hampering their use in humans, and is thought to be associated with PDE4D subtype inhibition. Preferential inhibition of PDE4B may maintain efficacy in treating pulmonary fibrosis whilst avoiding certain adverse events. In the present paper, the preclinical pharmacology of the novel PDE4 inhibitor BI 1015550 is characterized, with results suggesting that this compound has potential for the treatment of IPF and probably other fibrotic ILDs. The data also suggest that BI 1015550 could be an effective combination partner to nintedanib, either by complementary activities or by additive/synergistic effects.

## Materials and Methods

All assays performed comply with the recommendations on experimental design and analysis in pharmacology (Curtis et al., 2015).

### Recombinant Phosphodiesterase Activity *In Vitro* with Scintillation Proximity Assay

For the preparation of human recombinant PDEs of interest, the Bac-To-Bac baculoviral expression system from Invitrogen (Carlsbad, CA, United States) was used according to the instruction manual. In short, the full-length open reading frames of PDE1C (National Center for Biotechnology Information Gene-Ref-Seq identifier NG_051183), PDE3A (NG_030033), PDE4A (NG_029594, AA 156–886), PDE4C (NG_029629), PDE7A (NG_029614), and PDE9A (NG_047067) were cloned into the plasmid vector pFastbac. For PDE4B and PDE4D, active site fragments were used (PDE4B NG_029038: AA 152–484, PDE4D NG_027957, AA 79–438). The plasmids were transformed into DH10Bac bacteria, SF9 insect cells were transfected with the bacmid DNA, and the resulting baculoviruses were stored and used for further rounds of infections. For protein production, SF9 cells were infected until a cytopathic effect was visible (after about 72 h), the SF9 cells were harvested and broken up by 3 freeze/thaw cycles, and by shearing 10 times through a 0.1 mm cannula using a syringe. The cytoplasmic cell extract was separated by centrifugation (10 min, 14.000 × g, 4°C). The protein content was measured and an equal volume of 87% (v/v) glycerol was added prior to freezing at −20°C. The Phosphodiesterase Scintillation Proximity Assays (TRKQ7090 for [^3^H]cAMP, GE Healthcare Europe GmbH, Chalfont St Giles, United Kingdom, and TRKQ7100 for [^3^H]cGMP, Perkin Elmer, Waltham, MA, United States) were performed essentially according to the instruction manual. Briefly, serial dilutions of BI 1015550 were added to the assays [final dimethyl sulfoxide (DMSO) concentration of 1.1% (v/v)]. Assay solutions containing recombinant baculoviral-expressed PDE (to give an activity of 5.000–20.000 cpm corresponding to 5–20% of total activity added), 50 mmol/L Tris/HCl pH 7.5, 8.3 mmol/L MgCl_2_, 1.7 mmol/L EGTA, and 10 µL radiolabeled substrate (0.05 µCi in H_2_O) were incubated for 1 h at 30°C (final volume 100 μL). The reaction was stopped by addition of 50 µL of yttrium silicate scintillation proximity assay beads (17.8 mg/mL, 18 mM zinc sulfate in H_2_O) and the amount of produced [^3^H]AMP/[^3^H]GMP associated was determined using a Wallac Microbeta scintillation counter. For PDE2A, PDE5, and PDE6, inhibitory activity of BI 1015550 was screened at Eurofins Cerep, Celle L’Evescault, France.

### Tumor Necrosis Factor-α and Interleukin-2 Release in Human Peripheral Blood Mononuclear Cells

Blood was donated by internal donors at the Center for Occupational Health at Boehringer Ingelheim in Biberach an der Riss, Germany; the donors provided signed informed consent that allows use for scientific purposes. All research was performed in accordance with the principles stated in the Declaration of Helsinki. Ethical approval was provided by the Federal State Medical Association of Baden-Württemberg, reference no. F-2016–121. For preparation of peripheral blood mononuclear cells (PBMCs), 200 mL freshly drawn human blood from healthy donors was mixed with 50 mL acid-citrate-dextrose solution (38 mmol/L citric acid, 75 mmol/L tri-sodium citrate and 121 mmol/L glucose) and 50 mL Hanks buffered saline solution (Thermo Fisher Scientific, Carlsbad, CA, United States), overlaid on Ficoll and centrifuged for 30 min at 300 x g. PBMCs were diluted in RPMI-1640 medium (Thermo Fisher Scientific) containing 6% (v/v) autologous plasma to a concentration of 5 × 10^6^ cells/mL. PBMCs were incubated at 37°C, 5% CO_2_, and 95% humidity in the presence or absence of BI 1015550 and stimulated either with 100 ng/mL LPS (serotype 055:B5) (Sigma Aldrich, Deisenhofen, Germany) for 4 h (TNF-α assay), or with 10 µg/mL phytohemagglutinin P (Sigma Aldrich) for 20 h (IL-2 assay). Supernatants were taken and cytokines measured by enzyme-linked immunosorbent assay (ELISA; Becton Dickinson, Franklin Lakes, NJ, United States).

### Tumor Necrosis Factor-α and Interleukin-6 Release in Human and Rat Whole Blood

Heparinized whole blood was collected from the Aorta abdominalis of WI (Han) male rats (Charles River Laboratories, Sulzfeld, Germany) and from healthy male human donors, and treated with 1 µL (rat) or 2.5 µL (human) of BI 1015550 or vehicle [final DMSO concentration 0.5% (v/v)]. Final assay volumes were 200 μL (rat) and 500 μL (human). After incubation for 30 min at 37°C, 95% humidity, and 5% CO_2_, cultures were treated with LPS [final concentration 10 µg/mL (rat), 0.1 µg/mL (human)] or saline. After 7 h, plates were centrifuged at 3.200 × g at 4°C for 10 min and plasma was used for cytokine measurement. Meso Scale Discovery (MSD) (Rockville, MD, United States) rat and human pro-inflammatory panels were used for detection of TNF-α and IL-6 in rat plasma [diluted 1:2 (v/v)]) and human plasma [negative control undiluted, other samples diluted 1:200 (v/v)] according to the manufacturer’s instructions.

### Human Fibroblast Functions

Fibroblasts from donors with IPF (IPF-LF) (Asterand, Detroit, MI, United States) were grown in fibroblast basal medium (CC-3131, Lonza Walkersville, Inc., Walkersville, MD, United States) supplemented with FGM-2 SingleQuot Kit Supplements and Growth Factors (CC-4126, Lonza). Cells were grown in a humidified incubator at 37°C and 5% CO_2_. All assays were performed at passage 7 or 8. For assay set-up, cells were seeded in fibroblast growth medium plus supplements in assay-relevant densities. For TGF-β-stimulated assays, cells were seeded at 4,500 cells per well in 96-well plates. Proliferation assays were performed in 96-well plates at an initial seeding cell density on day 0 of 2,000 cells per well. After 24-h, the cell culture medium was changed to starvation medium (fibroblast basal medium without supplements). After a 24-h starvation period, cells were pre-incubated for 30 min with different concentrations of BI 1015550 plus/minus different concentrations of nintedanib and/or 1 nmol/L PGE_2_ and stimulated with the assay-relevant stimulus for the indicated time in the presence of the compound.

### α-Smooth Muscle Actin Western Blot Replacement Assay

For determination of α-SMA protein expression, IPF-LF cells were stimulated with TGF-β (4 ng/mL) for 48 h. Lysates were assayed in a Western blot replacement assay (MSD). In short, lysates were pipetted onto high binding plates (MSD) and were sequentially incubated with a mouse anti-human α-SMA antibody (Sigma Aldrich) and an anti-mouse sulfo-tag antibody (MSD). For analysis, an MSD sector imager was used.

### Gene Expression Assays (Taqman)

For determination of Col1A1, Col3A1, and FN mRNA expression, IPF-LF cells were stimulated with TGF-β (4 ng/mL) for 48 h. Cells-to-CT (Ambion, Thermo Fisher Scientific) lysate preparation was performed according to the instruction manual. Lysates were diluted 1:20 (v/v) in nuclease-free H_2_O. Quantitative real-time polymerase chain reactions were set up using the TaqMan™ Fast Advanced Master Mix (Applied Biosystems, Carlsbad, CA, United States) and species-specific gene expression assays: Col1A1 #Hs00164004_m1 FAM, Col3A1 #Hs0094309_m1 FAM, FN #Hs01549976_m1 FAM, 18S #4310893E. Gene expression analysis was performed with the ViiA7™ Real-Time PCR System and software (Applied Biosystems). Fold-change of gene expression was calculated by the Viia7 software (regulation based upon housekeeper).

### Proliferation Assay

IPF-LF cells were stimulated with bFGF (20 ng/mL) plus IL-1β (30 pg/mL). After 72 h of stimulation, 10-times concentrated bromodeoxyuridine was added and the cells were incubated for another 18 h (final concentration bromodeoxyuridine 10 μmol/L). Cell proliferation was assessed by bromodeoxyuridine assay (Roche, Mannheim, Germany) according to the instruction manual.

### Animal Models

All procedures used were as humane as possible and studies involving animals complied with the recommendations of ARRIVE (Kilkenny et al., 2010; Mcgrath et al., 2010). Animal care and experimental procedures complied with German and French national guidelines and legal regulations and were approved by the local ethical committees.

Animals were fed standard chow diet and tap water *ad libitum*, and housed in a controlled temperature (22°C), under a 12-h light–dark cycle. The group size was calculated by a statistician and defined in the respective permit.

### Lipopolysaccharide-Induced Tumor Necrosis Factor-α Release in Whole Blood *Ex Vivo* in Mice

BI 1015550 was suspended in H_2_O plus three equivalents’ HCl (i.e. 66 µL HCl per mg of drug substance) to concentrations of 0.03, 0.01, 0.001, and 0.0001 mg/mL. Female NMRI mice from Charles River (25–30 g) were used. Either vehicle (H_2_O plus three equivalents’ HCl) or BI 1015550 was administered orally (by gavage) in a volume of 10 mL/kg body weight resulting in final doses of 3.0, 1.0, 0.1, and 0.01 mg/kg body weight. 2 h after administration, the animals were anesthetized and blood was drawn by retroorbital puncture with heparinized capillaries before sacrifice by cervical dislocation. Blood was collected in Lithium-Heparin Microvettes® for anticoagulation. 100 µL of whole blood was pipetted into an Eppendorf tube containing either LPS solution (final concentration 900 ng/mL) or solvent. Subsequently, the samples were placed for 4 h in an incubator at 37°C, 0.5% CO_2_, and 95% humidity before plasma was prepared by a two-step centrifugation at 10.840 × g over 10 min at 4°C. TNF-α concentrations in the plasma samples were determined using a commercially available TNF-α ELISA kit (BD OptEIA™ Mouse TNF ELISA Set from Becton Dickinson) according to the manufacturer’s instructions.

### Lipopolysaccharide-Induced Neutrophil Influx Into the Bronchoalveolar Lavage Fluid of Male Suncus Murinus and Wistar Rats

Suncus murinus is phylogenetically closer to primates than rodents and has been shown to be quite sensitive to emesis induction by archetypal PDE4 inhibitors like rolipram and denbufylline (Sawanishi et al., 1997). Compared with other animal species used to study emesis induction by PDE4 inhibitors, like ferrets, dogs, minipigs, or monkeys, Suncus murinus allows the testing of a sufficient number of animals to provide a high statistical power. BI 1015550 or roflumilast was suspended in 0.5% Natrosol (hydroxyethylcellulose) (Merck, Darmstadt, Germany). Male Suncus murinus (weight 50–55 g) from Boehringer Ingelheim (Biberach an der Riss, Germany) or male Wistar rats (200**–**250 g, Charles River), eight animals per group, were used. Animals were pre-treated with oral BI 1015550 (doses of 0.1, 0.3, and 1.0 mg/kg) or roflumilast (doses 0.3, 1, and 3 mg/kg) 30 min before LPS exposure. For this purpose, animals in the positive control and treatment groups were put into a circular Plexiglas chamber with 16 pie-like compartments for one animal each, and were consecutively exposed to nebulized LPS for 30 min. The LPS solution used for nebulization contained 1 mg/mL LPS in phosphate-buffered saline (PBS). Nebulization was performed with the commercially available PARI Master® nebulizer with a PARI Master® LL adapter (Pari GmbH, Starnberg, Germany). 4 h after LPS exposure, the animals were anesthetized and euthanized by cervical dislocation. After sacrifice, the trachea was cannulated and bronchoalveolar lavage was performed by instilling and re-aspirating two-times 1 mL (Suncus murinus) or 5 mL (rat) lavage buffer (PBS +2% bovine serum albumin). BALF volumes were recorded manually. Determination of neutrophil numbers in BALF was performed using a blood hemacytometer (Sysmex XT-1800i, Kobe, Japan).

### Determination of Emesis in Male Suncus Murinus

All animals (50–55 g) were dosed orally by gavage immediately before transfer to whole-body plethysmography boxes. The volume of application was set to 10 mL/kg body weight. For assessment of the emetic events, animals were placed in the whole-body plethysmography boxes without any access to feed or water for 3 h. The whole-body plethysmography system used to detect emetic events consisted of five major components: 1) plethysmography boxes for mice (Buxco®, PLY 3211-F, Data Sciences International, New Brighton, MN, United States), 2) a pressure transducer with an amplifier, 3) a bias flow regulator, 4) a PC-based system to receive and process the amplified signals, and 5) a PC-based data acquisition system (NOTOCORD-hem™, Instem, Stone, United Kingdom) to process signals, as well as in-house-developed software to detect and identify changes in breathing pattern. Retching and vomiting induced characteristic changes in the breathing pattern of animals and these were recorded by whole-body plethysmography and translated for NOTOCORD recording. A number of preset definitions (computer-based criteria) characteristic for emetic events were determined. Peaks that met the set internal computer-based criteria and exceeded the threshold of more than 600 mV were labeled as emetic events by the system on a standard basis. The respective curves were consecutively displayed for individual user assessment whether the detected peak was an emetic event or not (plausibility check by technician).

### Bleomycin Mouse Model

Adult, test-naïve, male C57BL6/6J mice, aged 10–12 weeks from Charles River were used (weight 25–27 g). The model was performed essentially as described by Ackermann et al. (2017). Briefly, bleomycin 1 mg/kg (Calbiochem, Darmstadt, Germany) was administered intratracheally in an application volume of 2 mL/kg body weight. Animals were weighed daily. A body weight loss of 20% or more automatically resulted in euthanization. BI 1015550 (at final doses of 2.5 and 12.5 mg/kg) was applied by oral gavage using a dose volume of 10 mL/kg twice daily (b.i.d.) from day 8 until day 13. On day 12, animals were anesthetized using 3–4% isoflurane, and lung density was assessed by micro-computed tomography (µCT) analysis with a Quantum FX µCT system (Perkin Elmer) with cardiac gating (without respiratory gating). Images were analyzed using MicroView 2.0 software (GE Healthcare). The Hounsfield unit (HU) corresponding to the peak of the HU-histogram for the segmented pixels was used as a measure of fibrosis. After µCT analysis, animals were allowed to awaken from anesthesia. On day 14, lung function [pressure-volume loops, FVC, and static lung compliance (Cstat)] was measured with a FlexiVent system (SCIREQ, Montreal, PQ, Canada). After the lung function measurement, animals were sacrificed by an overdose of Narcorene and lungs were lavaged with two-times 0.8 mL of PBS. BALF was used to determine differential cell counts (Sysmex XT-1800iVet) and the number of monocytes. Left lung lobes were fixed with 4% paraformaldehyde and inflated under 20 cm water pressure for 20 min. Samples were embedded in paraffin, sectioned (3 μm thickness), and stained for hematoxylin and eosin (HE) and Masson’s trichrome to assess general morphology and fibrotic change following standard histopathology operating protocols. Images were taken with an AxioCam MRm microscope camera using AxioVision software (Carl Zeiss Microscopy, LLC, White Plains, NY, United States). Lung sections stained with Masson’s trichrome were assessed for severity of pulmonary fibrosis using the Ashcroft score. The protocol used is therapeutic, i.e. treatment commenced at a time where lung fibrosis should have developed, in line with the recommendations of the American Thoracic Society panel on IPF models (Jenkins et al., 2017).

### Silica-Induced Lung Inflammation and Fibrosis in the Mouse

BI 1015550 was given at doses of 0.25, 0.75, and 2.5 mg/kg b.i.d. in Natrosol 0.5% (10 mL/kg) starting from day 10 after silica instillation until day 30 (therapeutic regimen). 8-week-old BL6 mice (mixed sex, Janvier Labs, Le Genest-Saint-Isle, France) were used. Details of the experimental methods are described elsewhere (Lo Re et al., 2010). Briefly, mice received silica particles at 2.5 mg/mouse by intranasal instillation. Control mice received the respective saline solution by intranasal instillation. Mice were killed 30 days after silica administration and macroscopic changes were recorded at post-mortem analysis. Lung weight was measured, BALF for total cell and differential cell counts as well as myeloperoxidase (MPO) activity and keratinocyte-derived cytokine (KC, the counterpart of human GRO protein) determination was generated, and lung histology (staining with HE and chromotrope-aniline blue with semi-quantitative analysis for the estimation of inflammation, granuloma, and fibrosis scores was done.

### Data Analysis and Statistics

The data and statistical analyses comply with the recommendations on experimental design and analysis in pharmacology (Curtis et al., 2015).

All data are presented as means ± standard error of the mean (SEM) of n donors/animals. For the *in vitro* assays, technical replicates were used to ensure the reliability of single values. Unless otherwise noted, the statistical differences between groups were analyzed by one-way analysis of variance with subsequent Dunnett’s multiple comparison test for all parametric data and Kruskal–Wallis test followed by Dunn’s multiple comparison test for non-parametric data. Statistical significance was accepted at *p* < 0.05. The tests were performed using GraphPad Prism (GraphPad Software, La Jolla, CA, United States). To control for unwanted donor variances, the *in vitro* data were normalized by calculation of percentage inhibition according to: % inhibition = 100-(Y/K1)*100 (with K1 being the mean rate of non-stimulated, non-compound-treated control wells subtracted from the mean rate of stimulated, non-compound-treated control wells, and Y being the mean rate of non-stimulated, non-compound-treated control wells subtracted from the mean rate of stimulated, compound-treated wells). Inhibitory concentration for half-maximal inhibition (IC_50_) values were calculated by non-linear regression of log (inhibitor concentration) versus percentage inhibition using a three- or four-parameter fitting procedure of the GraphPad Prism software package. To calculate the additive effect of BI 1015550 combined with nintedanib, the following formula was used: effect of BI 1015550 (EB) + effect of nintedanib (EN) = EB + N = EB + EN−(EB*EN) (Pöch and Holzmann, 1980).

### Materials

BI 1015550, roflumilast, and nintedanib were synthesized at the chemical facilities of Boehringer Ingelheim (Biberach, Germany). Human recombinant TGF-β, bFGF and IL-1ß were purchased from R&D Systems, Inc. (Minneapolis, MN, United States). Human PGE_2_ was obtained from Tocris Bioscience (Bristol, United Kingdom).

## Results

### BI 1015550 Preferentially Inhibits Phosphodiesterase 4B

BI 1015550 preferentially inhibited hydrolysis of cAMP by PDE4B with an IC_50_ of 10 nmol/L, compared with 248 nmol/L for PDE4A, 8,700 nmol/L for PDE4C, and 91 nmol/L for PDE4D (Table 1). Under similar assay conditions, human PDE7A and human PDE3A were only weakly affected, with calculated IC_50_ values of 14 μmol/L and 120 μmol/L, respectively. Likewise, human PDE1C was only inhibited with high IC_50_ values (46 μmol/L and 85 μmol/L, respectively, using cAMP and cGMP as substrate), and the IC_50_ value for human PDE9A (substrate cGMP) was >100 μmol/L. BI 1015550 did not inhibit human PDE2A and PDE5, nor bovine PDE6.

**TABLE 1:** Inhibition of human recombinant PDE4 subtypes by BI 1015550 in comparison to roflumilast and its active metabolite Roflu-N-Ox. IC_50_ values (nmol/L) are given as means from *n* independent experiments. Cell extracts containing the active site fragments mediated the conversion of 10 µL [^3^H]cAMP (0.05 µCi in H_2_O) to AMP resulting in the binding of this radiolabeled molecule to the yttrium silicate SPA beads and the subsequent generation of scintillation events determined using a Wallac Microbeta counter. AMP, adenosine monophosphate; IC_50_, inhibitory concentration (nM) for half-maximal inhibition; PDE, phosphodiesterase; SPA, scintillation proximity assay.

	PDE4A	PDE4B2	PDE4C2	PDE4D2
BI 1015550	248 (*n* = 3)	10 (*n* = 2)	8,700 (*n* = 3)	91 (*n* = 2)
Roflumilast	1.4 (*n* = 2)	0.6 (*n* = 12)	12 (*n* = 2)	0.8 (*n* = 12)
Roflu-N-Ox	2.8 (*n* = 1)	1.4 (*n* = 6)	15 (*n* = 1)	1.4 (*n* = 6)

### BI 1015550 Inhibits Tumor Necrosis Factor-α and Interleukin-2 Release of Purified Human Peripheral Blood Mononuclear Cells

**FIGURE 1:**
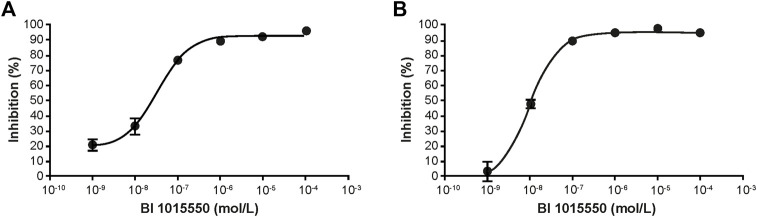
Inhibition of cytokine release in human PBMCs. TNF-α **(A)** and IL-2 **(B)** were induced by LPS and PHA-P, respectively. PBMCs were incubated at 37°C, 5% CO_2_, and 95% humidity in the presence or absence of BI 1015550 and stimulated either with 100 ng/mL LPS for 4 h (TNF-α assay), or with 10 µg/mL PHA-P for 20 h (IL-2 assay). Results are given as means ± SEM from 6 different donors. IL-2, interleukin-2; LPS, lipopolysaccharide; PBMC, peripheral blood mononuclear cell; PHA-P, phytohemagglutinin-P; SEM, standard error of the mean; TNF-α, tumor necrosis factor-α.

In human PBMCs, BI 1015550 inhibited LPS-induced TNF- α release with an IC_50_ of 35 nmol/L (Figure 1A), and inhibited phytohemagglutinin P-induced IL-2 release with an IC_50_ of 9 nmol/L (Figure 1B).

### BI 1015550 Inhibits Lipopolysaccharide-Induced Tumor Necrosis Factor-α Release in Human and Rat Whole Blood *In Vitro*, and Stimulates Interleukin-6 in Rat, but Not in Human, Whole Blood

**FIGURE 2:**
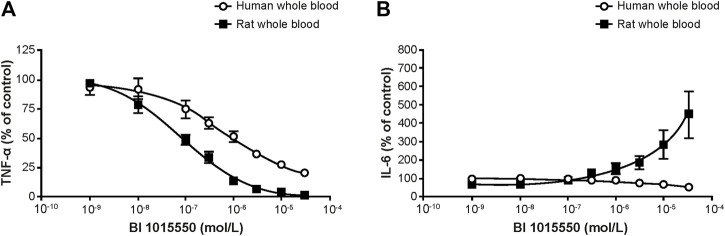
Effect of BI 1015550 on LPS-stimulated TNF-α **(A)** and IL-6 **(B)** release in human and rat whole blood. Results are given as means ± SEM from 4 independent experiments. Whole blood from WI (Han) male rats and from healthy male human donors was treated with 1 µL (rat) or 2.5 µL (human) of BI 1015550 or vehicle (DMSO 0.5% v/v). After incubation, cultures were treated with LPS at a final concentration of 10 µg/mL (rat), 0.1 µg/mL (human), or saline. DMSO, dimethyl sulfoxide; IL-6, interleukin-6; LPS, lipopolysaccharide; SEM, standard error of the mean; TNF-α, tumor necrosis factor-α; WI, Wistar.

In rat whole blood, BI 1015550 completely inhibited TNF-α release with an IC_50_ value of 91 nmol/L. In human whole blood, BI 1015550 inhibited TNF-α release up to 70–80% with an IC_50_ value of 670 nmol/L (Figure 2A). Under identical experimental conditions, high concentrations of BI 1015550 resulted in a fourfold concentration-dependent increase of IL-6 compared with LPS-treated samples without added PDE4 inhibitor in rat whole blood (Figure 2B). In contrast, in human whole blood, high concentrations of BI 1015550 did not result in an IL-6 increase, but rather resulted in a concentration-dependent reduction of IL-6 release with a maximum inhibition of about 30–40% (Figure 2B).

### BI 1015550 Inhibits Lipopolysaccharide-Induced Tumor Necrosis Factor-α Release in Whole Blood *Ex Vivo* in Mice

**FIGURE 3:**
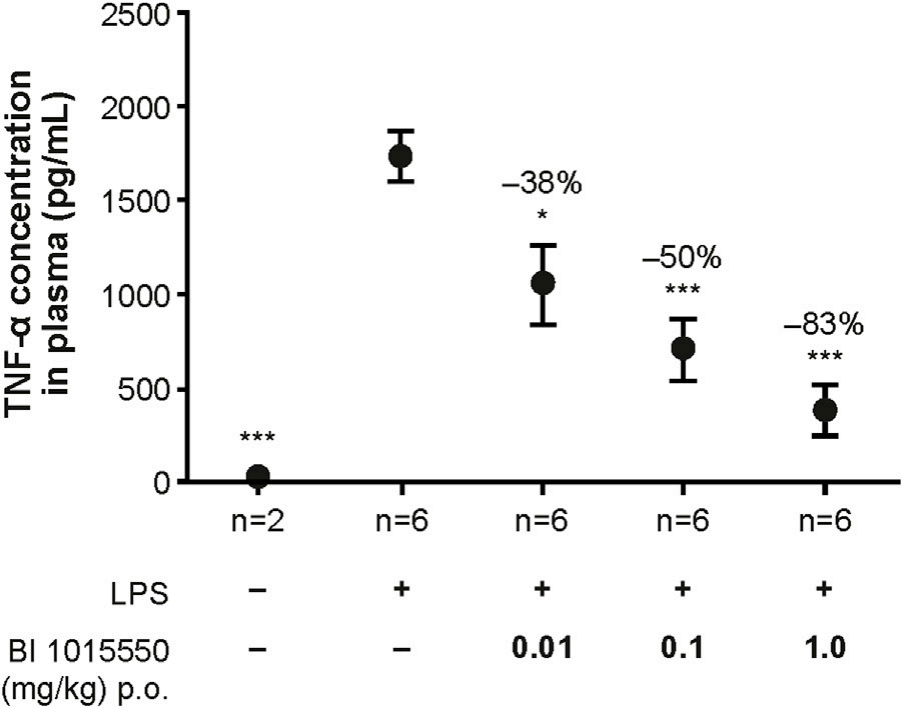
Inhibition of LPS-stimulated TNF-α release in whole blood *ex vivo* in mice by BI 1015550. Mice were pre-treated with solvent or oral BI 1015550 at the doses indicated for 2 h before blood sampling, and then whole blood was stimulated with LPS for 4 h. Inhibition of TNF-α in % is given as negative number. Results are shown as means ± SEM from the number of animals indicated. *, *p* < 0.05; ***, *p* < 0.001. LPS, lipopolysaccharide; p.o., orally; SEM, standard error of the mean; TNF-α, tumor necrosis factor-α.

In an initial experiment, BI 1015550 at 3 mg/kg inhibited TNF-α by 93% (data not shown). In a subsequent experiment, a dose–response relationship was shown when mice were treated with BI 1015550 at doses of 0.01, 0.1, and 1 mg/kg. The dose of BI 1015550 resulting in half-maximal inhibition (ED_50_) of LPS-induced TNF-α release was determined to be 0.04 mg/kg (Figure 3).

### BI 1015550 Inhibits Lipopolysaccharide-Induced Neutrophil Influx Into the Bronchoalveolar Lavage Fluid of Male Suncus Murinus and Wistar Rats

To directly compare efficacy and tolerability, the *in vivo* activity of BI 1015550 was assessed in the BALF of Suncus murinus exposed to nebulized LPS.

**FIGURE 4:**
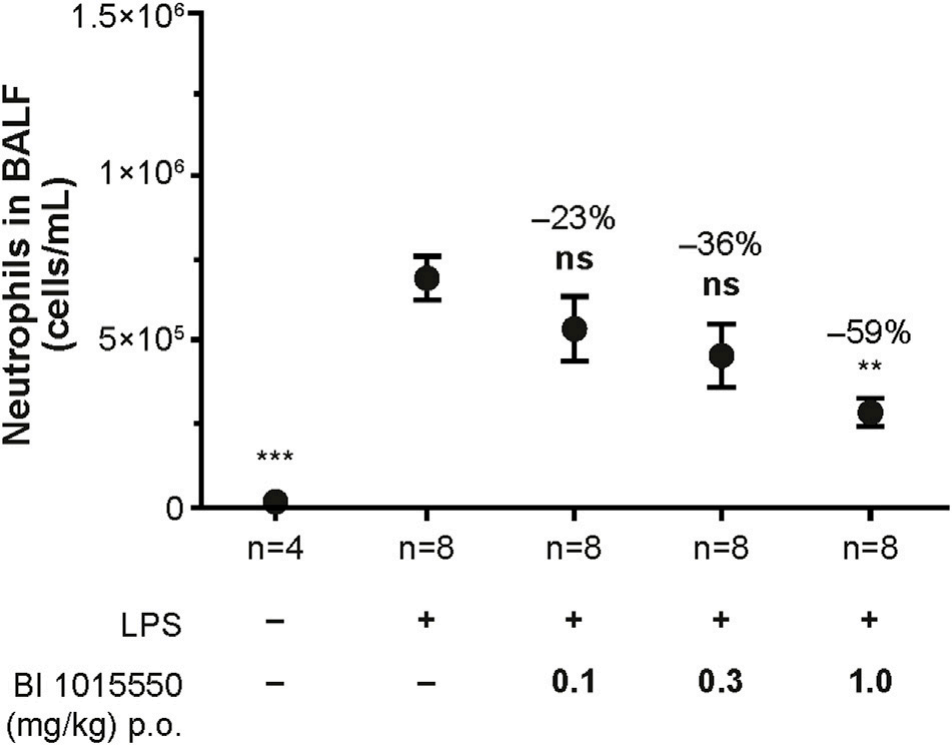
Inhibition of LPS-induced neutrophil influx into the BALF of Suncus murinus by BI 1015550. Animals were pre-treated with oral BI 1015550 at the doses indicated for 30 min before exposure to nebulized LPS for a further 30 min. Thereafter, BALF was prepared and neutrophils were counted. Inhibition of neutrophils in % is given as negative number. Results are shown as means ± SEM from the number of animals indicated. **, *p* < 0.01; ***, *p* < 0.001. BALF, bronchoalveolar lavage fluid; LPS, lipopolysaccharide; ns, not significant; p.o., orally; SEM, standard error of the mean.

Untreated control animals not exposed to LPS exhibited only a very low number of neutrophils in the BALF. Exposure to LPS led to a strong influx of neutrophils into the BALF. Treatment of the animals with BI 1015550 at doses of 0.1, 0.3, and 1 mg/kg led to a dose-dependent inhibition of the LPS-induced neutrophil influx into the BALF. The ED_50_ calculated was 0.6 mg/kg (Figure 4). Roflumilast (0.3, 1, and 3 mg/kg) was used as a reference (ED_50_ = 1 mg/kg).

BI 1015550 (doses 0.01, 0.1, and 1 mg/kg) and roflumilast (doses 0.3, 1, and 3 mg/kg) inhibited LPS-induced lung neutrophil influx in male Wistar rats with an ED_50_ of 0.1 mg/kg and 1 mg/kg, respectively (data not shown).

### BI 1015550 Has a Low Emetic Potential in Male Suncus Murinus

After administration of BI 1015550 at a dose of 0.5 mg/kg (close to the ED_50_ determined in the neutrophil influx model), 3 of 24 animals tested showed emesis, with 0.1 mean emetic events per animal (data not shown). With 6 mg/kg BI 1015550 (∼10 times the ED_50_ determined in the neutrophil influx assay), 5 out of 24 (21%) animals showed emesis, with 0.3 mean emetic events per animal (Table 2). With roflumilast at 10 mg/kg (10x ED_50_), emesis was induced in 10/24 animals (42%, with a mean of 0.7 events per animal). The emetic potential of BI 1015550 was comparable to untreated animals (2 out of 24 tested animals showed emesis, with 0.1 mean emetic events per animal) and animals treated with vehicle (0.5% Natrosol) (3 out of 24 tested animals showed emesis, with 0.1 mean emetic events per animal).

**TABLE 2:** Comparison of oral BI 1015550 and roflumilast in Suncus murinus with respect to pharmacodynamic efficacy (inhibition of LPS-induced neutrophils in BALF) and emesis induction. Data for roflumilast were obtained under identical conditions as described for BI 1015550. ED_50_ of roflumilast was determined with doses of 0.3, 1, and 3 mg/kg and 8 animals per group; emesis was estimated with 24 animals for both compounds. BALF, bronchoalveolar lavage fluid; ED_50_, effective dose for half-maximal inhibition; LPS, lipopolysaccharide; n. a., not assessed.

	Natrosol	BI 1015550	Roflumilast
**LPS-induced BALF neutrophils**			
ED_50_ (mg/kg)	n.a.	0.6	1.0
**Emesis at 10 × ED_50_**			
Mean events per animal	0.1	0.3	0.7
% of vomiting animals	13	21	42

### BI 1015550 is Active in the Therapeutic Bleomycin Model in Mice

Typically in this model, animals lose body weight approximately 3 days after bleomycin administration, but then gain weight at a normal rate from day 8 onwards. There was no significant effect of BI 1015550 treatment on weight gain (data not shown).

**FIGURE 5:**
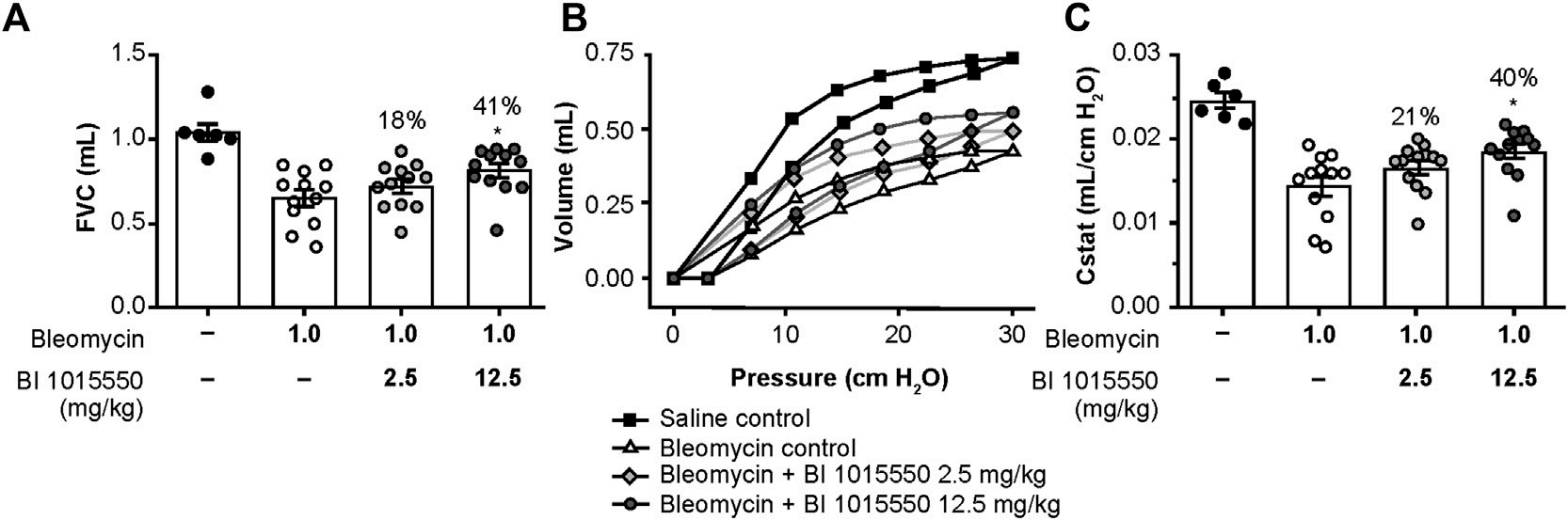
Effects of oral BI 1015550 (2.5 and 12.5 mg/kg b.i.d.) on bleomycin-challenged mice on day 14. Inhibition of **(A)** FVC decline, **(B)** PV loops, and **(C)** lung volume (Cstat) at 30 cm H_2_O. Bleomycin was administered intratracheally in an application volume of 2 mL/kg body weight. BI 1015550 (in 0.5% hydroxyethylcellulose) was administered via oral gavage in a dose volume of 10 mL/kg b.i.d., from day 8 to day 13. Data are expressed as means ± SEM. *, *p* < 0.05. b.i.d., twice daily; Cstat, static lung volume; FVC, forced vital capacity; PV, pressure-volume; SEM, standard error of the mean.

Following bleomycin challenge, there was a decrease in FVC. The lower dose of BI 1015550 (2.5 mg/kg) induced a small numerical, but non-significant improvement. However, the higher dose of BI 1015550 (12.5 mg/kg b.i.d.) was associated with a statistically significant improvement in FVC of 41% (*p* < 0.05) (Figure 5A; Table 3).

**TABLE 3:** Effects of BI 1015550 in the mouse bleomycin model. Results are given as % improvement by the indicated BI 1015550 doses administered b.i.d. compared to bleomycin control. Bleomycin was administered intratracheally in an application volume of 2 mL/kg body weight. BI 1015550 (in 0.5% hydroxyethylcellulose) was administered via oral gavage in a dose volume of 10 mL/kg b.i.d., from day 8 to day 13. *, *p* < 0.05 based on adjusted covariant analysis (ANCOVA). ANCOVA, analysis of covariance; b.i.d., twice daily; Cstat, static lung compliance; FVC, forced vital capacity.

	FVC (mL)	Cstat (mL/cm H_2_O)	Fibrosis < score	Ratio tissue density
2.5 mg/kg	18	21	2	4
12.5 mg/kg	41*	40*	11	39

Following bleomycin challenge, there was an impairment in the pulmonary pressure-volume (PV) loops. The lower dose of BI 1015550 induced a small numerical, but non-significant improvement in PV loops. However, again the higher BI 1015550 dose was associated with a statistically significant improvement in PV loops of 40% (*p* < 0.05) (Figure 5B). The calculated Cstat at a pressure of 30 cm/H_2_O was similarly improved (Figure 5C; Table 3).

Administration of bleomycin significantly increased lung tissue density assessed by µCT, whereas treatment with BI 1015550 at the higher dose numerically reduced the ratio of dense fibrotic tissue to total lung volume by 39% (Table 3), although this was not statistically significant compared with untreated animals.

### BI 1015550 is Active in a Therapeutic Murine Model of Progressive Lung Fibrosis Induced by Silica Particles

A single intranasal administration of silica resulted in robust lung inflammation, with a marked increase in total cells, macrophages, neutrophils, and lymphocytes in the BALF, as well as several pro-inflammatory mediators like MPO or KC. BI 1015550 was administered orally in a therapeutic regimen (day 10–30) at doses of 0.25, 0.75, and 2.5 mg/kg, and the results are summarized in Table 4. BI 1015550 dose-dependently improved microscopic scores for granuloma formation, fibrosis, and inflammation, although these parameters did not reach statistical significance. In BALF, the highest dose of BI 1015550 (2.5 mg/kg) inhibited macrophages and neutrophils (*p* < 0.5) among the cells investigated. Other BALF parameters (MPO and KC) were also inhibited substantially at the higher doses (0.75 and 2.5 mg/kg). However, these effects again did not reach statistical significance. Lung weight was reduced by BI 1015550, although this effect was not dose dependent.

**TABLE 4:** Effects of BI 1015550 in the mouse model of silica-induced lung fibrosis. Results are given as % change related to control animals. Each treatment group contained 10 animals. Negative values indicate a reduction of the parameter indicated. Lung histology was done semi-quantitatively after hematoxylin and eosin and chromotrope-aniline blue staining (see scores). Cell counts, MPO, and KC were determined in BALF. *, *p* < 0.05. BALF, bronchoalveolar lavage fluid; KC, keratinocyte-derived cytokine; MPO, myeloperoxidase.

	0.25 mg/kg	0.75 mg/kg	2.5 mg/kg
Granuloma score	–16	–11	–32
Fibrosis score	5	–10	–15
Inflammation score	–4	4	–19
Total cells	–27	–13	–28
Macrophages	–29	–11	–38*
Neutrophils	–35	8	–46*
Lymphocytes	–18	49	–12
MPO	–2	–22	–34
KC	4	–39	–27
Lung weight	–57	–46	–46

### BI 1015550 Shows Complementary Activity on Human Myofibroblast Transformation Compared with Nintedanib and a Synergistic Effect in Combination with Nintedanib on Fibroblast Proliferation

**FIGURE 6:**
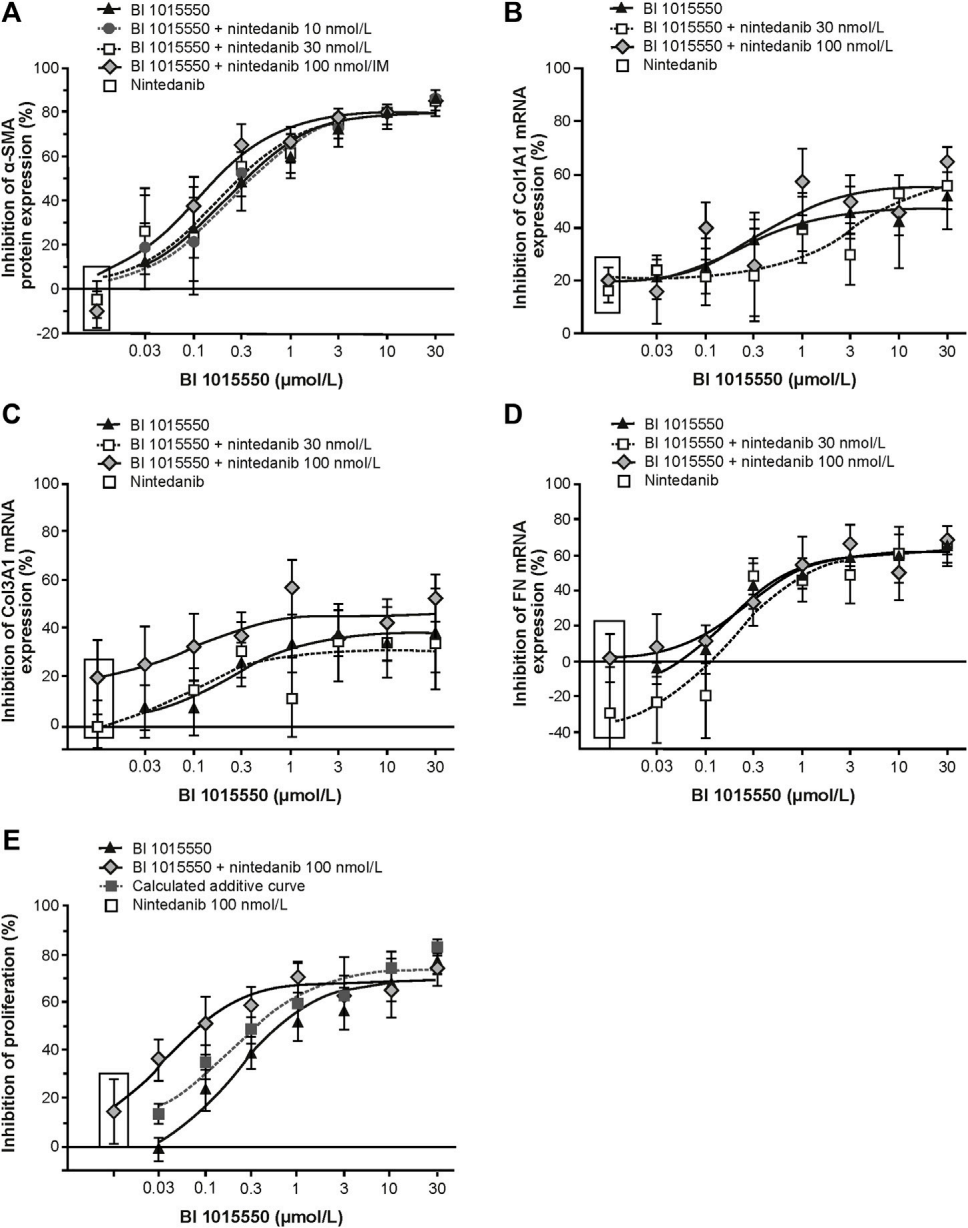
Inhibition of primary lung fibroblasts from patients with IPF by BI 1015550. **(A)** TGF-ß-stimulated transformation into myofibroblasts (*n* = 6); TGF-ß-stimulated mRNA expression of Col1A1 **(B)**, Col3A1 **(C)**, and FN **(D)** (*n* = 6, each); **(E)** IL-1ß plus FGF-induced proliferation (*n* = 5). Fibroblasts from donors with IPF were pre-incubated with different concentrations of BI 1015550 plus/minus different concentrations of nintedanib and/or 1 nmol/L PGE_2_. Results are given as means ± SEM from the number of donors indicated. α-SMA, α-smooth muscle actin; Col, collagen type; FGF, fibroblast growth factor; FN, fibronectin; IL-1ß, interleukin-1ß; IPF, idiopathic pulmonary fibrosis; PGE_2_, prostaglandin E_2_; SEM, standard error of the mean; TGF-ß, transforming growth factor-ß.

BI 1015550 inhibited α-SMA protein expression of TGF-β-stimulated IPF-LF with an IC_50_ of 210 nmol/L. Combination with nintedanib 10–100 nmol/L did not result in additional inhibitory efficacy (Figure 6A). Nintedanib alone up to the highest concentration of 100 nmol/L had no inhibitory effect on α-SMA protein expression. BI 1015550 attenuated TGF-β-induced Col1, Col3, and FN mRNA expression, with IC_50_ values of 269, 213, and 246 nmol/L, respectively (Figures 6B–D). The combination with nintedanib at 100 nmol/L showed additive effects in inhibiting Col3 mRNA expression (Figure 6C). BI 1015550 inhibited bFGF plus IL-1β-induced cell proliferation with an IC_50_ of 255 nmol/L. Nintedanib (100 nmol/L) alone inhibited proliferation by 15%. The combination of BI 1015550 plus 100 nmol/L nintedanib resulted in synergistic inhibitory effects and shifted the concentration–response curve to the left towards an IC_50_ of 23 nmol/L (Figure 6E). The combination of BI 1015550 with pirfenidone (100 µmol/L) did not yield any additional inhibitory effects in the two described fibroblast *in vitro* assays used (data not shown).

## Discussion

The present paper describes the preclinical pharmacology of BI 1015550, a novel oral PDE4 inhibitor that preferentially inhibits PDE4B.

Among the large superfamily of PDE isoenzymes, BI 1015550 selectively inhibits PDE4, and so far, has not shown any other off-target effects in screening against a panel of enzymes and receptors (Cerep high-throughput profile and Cerep non-kinase enzyme profile, Euriofins Cerep, Celle L’Escevault, France). Compared with roflumilast, the only oral PDE4 inhibitor on the market for a lung indication, BI 1015550 shows a unique profile in inhibiting PDE4 subtypes by a preferential inhibition of PDE4B (Table 1). The use of earlier PDE4 inhibitors, including roflumilast and apremilast, has been hampered by gastrointestinal side effects such as nausea, emesis, and/or diarrhea in humans. Although not proven, there is some evidence that these side effects are linked to inhibition of the PDE4D subtype (Giembycz, 2002). In this respect, the roughly 10-fold selectivity of BI 1015550 for inhibition of PDE4B versus PDE4D may be of clinical importance. It may also explain why in Suncus murinus the therapeutic ratio (inhibition of LPS-induced neutrophil influx into the lung versus induction of emesis) of BI 1015550 is superior to roflumilast (Figure 4; Table 2). More importantly, ongoing phase I clinical trials with BI 1015550 support the excellent gastrointestinal tolerability and safety of this compound in humans (data not shown). Another aspect of the use of BI 1015550 in humans relates to toxicity evident in animal studies. The major toxicity of PDE4 inhibitors, especially in the rat, is the induction of a vasculopathy (vasculitis, arteritis) in different tissues, which in some ways is paradoxical given that PDE4 inhibition is strongly linked to anti-inflammatory efficacy (see below). Although induction of vasculitis has never been reported in patients for the two oral PDE4 inhibitors roflumilast and apremilast, which have been on the market for many years, there is still some concern on this potentially serious side effect for the introduction of new PDE4 inhibitors. Dietsch et al. were the first to report stimulation of IL-6 in the rat by the PDE4 inhibitor IC542 (Dietsch et al., 2006). Since IL-6 is a possible candidate for induction of vasculitis in the rat, the lack of stimulatory effect of BI 1015550 on IL-6 in the human setting (Figure 2B) may indicate that, in contrast to rats, the risk for vasculitis induction by BI 1015550 in humans may be quite low. In addition, it is interesting to note that apremilast was reported to be clinically active in Behcet’s disease, a form of vasculitis characterized by inflammation of blood vessels, by resolution of oral ulcers, one of the most common symptoms (Hatemi et al., 2015).

Despite the alleviated inhibition of the PDE4D subtype, the well-known anti-inflammatory and immune-modulating properties of PDE4 inhibitors are still evident for BI 1015550. Qualitatively similar to roflumilast (Hatzelmann and Schudt, 2001), BI 1015550 appeared to be a potent inhibitor of TNF-α and IL-2 in human PBMCs (Figure 1), which, most likely, reflects the impact of BI 1015550 on monocytes and T lymphocytes, respectively. The efficient inhibition of TNF-α was also evident in whole blood (Figure 2), which is of importance because this assay format (*ex vivo*) can be used as a pharmacodynamic biomarker during clinical studies, as shown first by Timmer et al. for roflumilast (Timmer et al., 2002). Although the higher IC_50_ value of BI 1015550 for TNF-α inhibition in human whole blood (670 nmol/L) compared with human PBMCs (35 nmol/L) may be explained partly by the moderate plasma protein binding of the compound (77%), the loss of potency is higher than expected. In an effort to mimic the clinical situation, the potent and efficient inhibition of LPS-stimulated TNF-α in whole blood *ex vivo* was demonstrated for the mouse model in this study (Figure 3). It is clear that the ED_50_ of oral BI 1015550 (0.04 mg/kg) in this mouse model was much lower than in the mouse lung fibrosis disease models (bleomycin, silica) discussed below. However, in the former *ex vivo* model, blood is taken close to the maximal plasma concentration of the compound, and, although TNF-α as the pharmacologic read-out may contribute to lung fibrosis pathology, it is not the only relevant mediator. The efficacy and potency of BI 1015550 in the disease models will depend on other pathophysiologic aspects, and on the pharmacokinetics and availability of the compound during 24 h, which will be below the maximal plasma concentration due to the rather short half-life of the compound.

The aberrant wound-healing process in lung fibrosis passes through an inflammatory phase, with the involvement of inflammatory cells (in particular macrophages, monocytes, neutrophils, and T lymphocytes) and increased levels of cytokines (e.g. TNF-α and IL-1ß) and growth factors (e.g. TGF-ß and CTGF), creating a biochemical environment that supports chronic tissue remodeling and fibrosis. Based on this working hypothesis of the pathophysiology of lung fibrosis, the anti-inflammatory (inhibition of TNF-α, inhibition of monocytes, inhibition of neutrophil influx into the lung) and immune-modulatory (inhibition of IL-2, inhibition of T cells) characteristics of BI 1015550 can be expected to contribute to improvement of fibrosis, at least to some extent. Therefore, we were interested to see whether BI 1015550 exerts antifibrotic effects in distinct animal models, and further, if the compound has direct effects on fibroblasts and myofibroblasts.

Bleomycin has been widely used in rodents to model pulmonary fibrosis, to understand mechanisms involved in fibrogenesis, and to evaluate potential new therapies. However, it should be noted that although bleomycin-induced pulmonary fibrosis mimics many features of human disease, fibrosis resolves in rodents whereas in humans it is generally irreversible. Furthermore, whilst human pulmonary fibrosis predominantly impacts the peripheral airways, in rodents it is often found more prominently around the central airways. Despite these restrictions, the bleomycin model is still a good tool to assess the efficacy of potential compounds in general as proof of principle (Moeller et al., 2008; Jenkins et al., 2017). The therapeutic treatment regimen of BI 1015550 attenuated some important aspects of disease pathology, including FVC and Cstat decline (Figure 5). These therapeutic effects were statistically significant at the highest BI 1015550 dose. In addition, lung tissue density was improved to the same extent, although this effect did not reach statistical significance (Table 3). With regards to the Ashcroft score, BI 1015550 only showed a non-significant trend towards inhibition. For other experimental parameters investigated (BALF protein and monocytes, airway compliance), no effects of BI 1015550 were detected. The lack of effect of BI 1015550 on inflammatory mediators in BALF as well as the minimal effects on histologically assessed lung fibrosis in the present study are in contrast to bleomycin mouse studies reported by others (Cortijo et al., 2009; Udalov et al., 2010). We can only speculate that fibrotic changes in the mouse bleomycin model performed under our conditions are not captured by our scoring system, originally designed to assess human pulmonary fibrosis. It might also be possible that the changes in tissue volume do not only reflect fibrotic changes, but are related to other remodeling aspects like neovascularization in our model, as shown by Ackermann et al. (Ackermann et al., 2017). Differences versus previous studies may also be due to differing treatment durations and timings of data collection. Regardless of the nature of such changes, improvement in functional parameters, such as lung function, suggests a positive impact of BI 1015550 in pulmonary tissue remodeling. This assumption is supported by the positive findings for BI 1015550 in the silica-induced lung fibrosis model in the mouse. In contrast to bleomycin, which induces reversible lung fibrosis, silica particles induce a progressive type of lung fibrosis, which resembles the human counterpart of a progressive fibrosing ILD. To the best of our knowledge, a PDE4 inhibitor that preferentially inhibits PDE4B has not been evaluated in this animal model before. As shown in Table 4, BI 1015550 in a therapeutic regimen achieved dose-dependent improvements in semi-quantitative scores of granuloma formation, inflammation, and fibrosis, although these effects did not reach statistical significance. In BALF, macrophages and neutrophils were significantly inhibited at the highest dose, while the inhibition of MPO and KC did not reach statistical significance.

Overall, the beneficial effects of BI 1015550 in two lung fibrosis models are in agreement with preclinical data in the literature that selective PDE4 inhibitors could be clinically effective not only in lung fibrosis but, based on animal data, probably also in fibrosis of other organs (e.g. liver, kidney, colon, and/or skin). This hypothesis is supported by several studies showing that selective PDE4 inhibitors are able to directly target fibroblasts in addition to inflammatory and immune-competent cells. By using relevant cells, namely human lung fibroblasts from patients with IPF, we were able to support this by showing that BI 1015550 inhibits TGF-ß-stimulated transformation into myofibroblasts (Figure 6A). This indicates a differential mode of action for BI 1015550 compared with nintedanib, which was inactive under these conditions and did not enhance the efficacy of BI 1015550. Furthermore, BI 1015550 attenuated TGF-β-induced Col1, Col3, and FN gene expression (Figures 6B–D), which are important constituents of fibrotic extracellular matrix, and the combination with nintedanib at 100 nmol/L showed additive effects in inhibiting Col3 expression. In addition, BI 1015550 inhibited bFGF plus IL-1β-induced cell proliferation. Nintedanib by itself, at a relevant concentration (100 nmol/L), inhibited the proliferation by only 15%, but, most strikingly, the combination of BI 1015550 plus nintedanib resulted in synergistic inhibitory effects and shifted the concentration–response curve to the left by about 10-fold (Figure 6E). Inhibition of human lung fibroblast proliferation and myofibroblast transformation *in vitro* might contribute to potential clinical efficacy of BI 1015550 in IPF. Furthermore, the combination of BI 1015550 with nintedanib might yield synergistic therapeutic effects in patients with IPF and other fibrotic lung diseases. This kind of synergism between a PDE4 inhibitor that preferentially inhibits PDE4B and nintedanib has not been reported before.

In summary, BI 1015550 is an oral preferential inhibitor of PDE4B with suggested improved tolerability in humans compared with the selective oral PDE4 inhibitors on the market; the preclinical profile suggests that this compound is a promising oral clinical drug candidate for the treatment of IPF and other ILDs. A phase II trial of BI 1015550 versus placebo in patients with IPF is currently ongoing (NCT04419506). Future clinical studies will show whether BI 1015550 will be able to provide substantial efficacy alone, or whether the combination with IPF standard therapies such as nintedanib may be preferred in order to fully exploit the therapeutic potential of BI 1015550.

## Data Availability

The raw data supporting the conclusion of this article will be made available by the authors, without undue reservation.

## Glossary

µCT: micro-computed tomography
AA: amino acid
b.i.d.: twice daily
BALF: bronchoalveolar lavage fluid
bFGF: basic fibroblast growth factor
cAMP: cyclic adenosine monophosphate
Col: collagen type
COPD: chronic obstructive pulmonary disease
Cstat: static lung compliance
CTGF: connective tissue growth factor
DMSO: dimethyl sulfoxide
EB: effect of BI 01015550
**ED**_**50**_: effective dose for half-maximal inhibition
ELISA: enzyme-linked immunosorbent assay
EN: effect or nintedanib
FN: fibronectin
FVC: forced vital capacity
h: hour(s)
HE: hematoxylineosin
HU: Hounsfield unit
**IC**_**50**_: inhibitory concentration for half-maximal inhibition
IL: interleukin
ILD: interstitial lung disease
IPF: idiopathic pulmonary fibrosis
KC: keratinocyte-derived cytokine
LPS: lipopolysaccharide
min: minute(s)
MPO: myeloperoxidase
PBMCs: peripheral blood mononuclear cells
PBS: phosphate-buffered saline
PDE: phosphodiesterase
**PGE**_**2**_: prostaglandin E_2_
PV: pressure-volume
SEM: standard error of the mean
SSc: systemic sclerosis
TGF-ß: transforming growth factor-ß
TNF-α: tumor necrosis factor-α
α-SMA: α-smooth muscle actin
