# Corrigendum: BI 1015550 is a PDE4B inhibitor and a clinical drug candidate for the oral treatment of idiopathic pulmonary fibrosis

**DOI:** 10.3389/fphar.2023.1219760

**Published:** 2023-05-30

**Authors:** Franziska Elena Herrmann, Christian Hesslinger, Lutz Wollin, Peter Nickolaus

**Affiliations:** Boehringer Ingelheim Pharma GmbH & Co. KG, Biberach an der Riss, Germany

**Keywords:** PDE4B, IPF, lung fibrosis, phosphodiesterase, cAMP, ILDs

In the original article, there was an error in Table 1 as published. The *n* numbers given in the legend to Table 1 were only applicable to the first row of the table. The corrected Table 1 and its caption appear below.

**TABLE 1:** Inhibition of human recombinant PDE4 subtypes by BI 1015550 in comparison to roflumilast and its active metabolite Roflu-N-Ox. IC_50_ values (nmol/L) are given as means from *n* independent experiments. Cell extracts containing the active site fragments mediated the conversion of 10 µL [^3^H]cAMP (0.05 µCi in H_2_O) to AMP resulting in the binding of this radiolabeled molecule to the yttrium silicate SPA beads and the subsequent generation of scintillation events determined using a Wallac Microbeta counter. AMP, adenosine monophosphate; IC_50_, inhibitory concentration (nM) for half-maximal inhibition; PDE, phosphodiesterase; SPA, scintillation proximity assay.

	PDE4A	PDE4B2	PDE4C2	PDE4D2
BI 1015550	248 (*n* = 3)	10 (*n* = 2)	8,700 (*n* = 3)	91 (*n* = 2)
Roflumilast	1.4 (*n* = 2)	0.6 (*n* = 12)	12 (*n* = 2)	0.8 (*n* = 12)
Roflu-N-Ox	2.8 (*n* = 1)	1.4 (*n* = 6)	15 (*n* = 1)	1.4 (*n* = 6)

The authors apologize for this error and state that this does not change the scientific conclusions of the article in any way. The original article has been updated.

